# Longevity

**Published:** 1860-07

**Authors:** 


*Longevity.—Beauly v. Roseneath.*—A correspondent writes: "It being questioned in reference to Roseneath whether any Highland parish could produce four living persons whose united ages amount to 370 years, I have to state that there are alive in the parish of Kilmorack three females and one male whose united ages amount to 375 years, being respectively 99, 94, 91, 91. The two eldest are widows, the others a widower and a spinster. This gives an average of 1 1-4 above Roseneath. It cannot be vouched for that these four are the eldest in the parish, and lately much higher figures could be brought forward, as no doubt many other Highland parishes can at present."—*Inverness Advertiser.*

